# Canakinumab leads to rapid reduction of neutrophilic inflammation and long-lasting response in Schnitzler syndrome

**DOI:** 10.3389/fmed.2023.1050230

**Published:** 2023-03-15

**Authors:** Simon Bossart, S. Morteza Seyed Jafari, Kristine Heidemeyer, Kexiang Yan, Laurence Feldmeyer, Luca Borradori, Nikhil Yawalkar

**Affiliations:** ^1^Department of Dermatology, Inselspital, Bern University Hospital, University of Bern, Bern, Switzerland; ^2^Department of Dermatology, Huashan Hospital, Fudan University, Shanghai, China

**Keywords:** Canakinumab, Schnitzler syndrome, neutrophilic inflammation, immunohistochemical, autoinflammatory disorders

## Abstract

Interleukin-1 (IL-1)-blocking therapies are effective in reducing disease severity and inflammation in Schnitzler syndrome. Here, we present a patient with Schnitzler syndrome treated successfully using canakinumab for over 10 years. Complete clinical response was associated with a decrease in dermal neutrophil number and expression of the pro-inflammatory cytokines IL-1β, IL-8, and IL-17 as assessed by immunohistochemical studies.

## Introduction

Schnitzler syndrome is a rare autoinflammatory disorder, defined by an urticarial rash associated with a monoclonal immunoglobulin (Ig) M gammopathy and at least two additional minor criteria, such as fever, arthralgia, lymphadenopathy, hepatomegaly, splenomegaly, increased markers of systemic inflammation, or abnormal findings on bone imaging (1). Approximately 20% of all cases with Schnitzler syndrome develop a lymphoproliferative disorder, such as Waldenström macroglobulinemia (1, 2). Treatment options for Schnitzler’s syndrome are limited and include nonsteroidal anti-inflammatory drugs, fluoroquinolones, oral glucocorticoids, and immunosuppressive drugs, which often fail to provide long-term remission and have safety issues (1). Ample evidence exists indicating a critical role of the interleukin-1 (IL-1) pathway in the pathogenesis of Schnitzler’s syndrome, which is substantiated by the beneficial response with an IL-1 blockade (3). Here, we present a case with excellent long-term response to canakinumab, which was associated with a rapid decrease in neutrophils and pro-inflammatory cytokines.

## Case report

**Figure 1:**
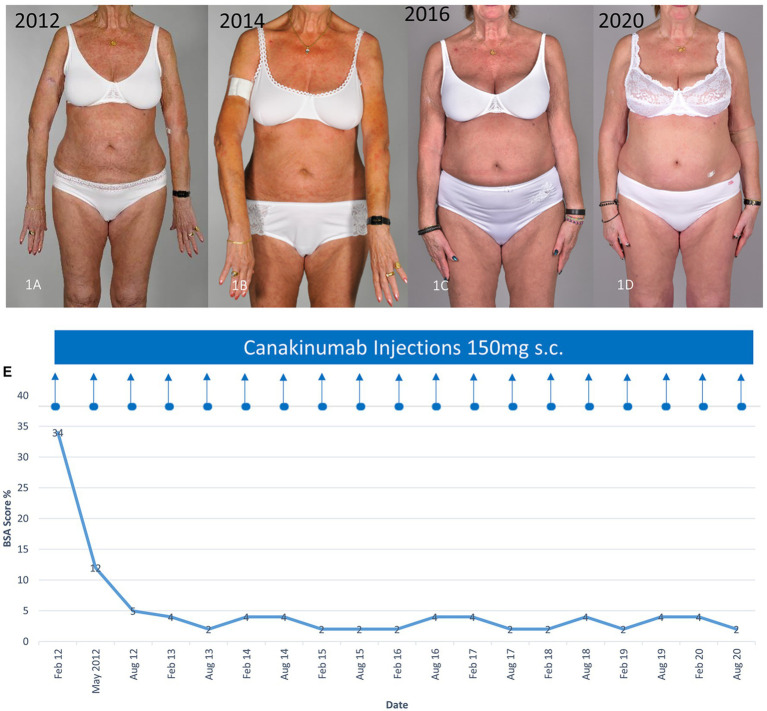
Clinical improvement of the characteristic urticarial eruption using canakinumab. **(A)** Before canakinumab, **(B–D)** during therapy with canakinumab 150 mg every 6 months **(E)** Clinical response assessed using the body surface area (BSA). The time course of the treatment with canakinumab and BSA affected with the urticarial rash are shown.

A 71-year-old woman presented with eruptions consisting of rose to red macules and slightly raised plaques affecting the trunk and limbs as well as bone pain, recurrent fever, night sweats, and weight loss in recent years. Light microscopy studies of a skin biopsy specimen (Hematoxylin and eosin staining) revealed a mixed inflammatory cellular dermal and perivascular infiltrate of lymphocytes and neutrophils. Extensive work up with serum protein electrophoresis and immunofixation as well as a bone marrow biopsy revealed an IgM monoclonal gammopathy of undetermined significance (MGUS; serum IgM monoclonal protein, 12.8 g/l). Blood tests showed elevated systemic inflammatory signs: high erythrocyte sedimentation rate (ESR), 55 mm/h; C-reactive protein (CRP), 30 mg/l (range < 5 mg/l); and leukocytes, 9 G/l (range 3.5–10.5 G/l). Based on these clinical and laboratory findings, the diagnosis of Schnitzler syndrome was made. The disease was controlled using oral prednisolone (1 mg/kg body weight daily). However, attempts to reduce the doses to 20 mg or less led to recurrences of the rash, fever, and bone pain. Further regimens including colchicine and pefloxacine were also ineffective to control the clinical manifestations. Therefore, canakinumab 150 mg subcutaneously was initiated, which led to a rapid improvement of the symptoms within 24 h. The extent of involvement calculated using the body surface area (BSA), reduced from 34 to 12% after 3 months of therapy (Figures 1A–E). Hematological and inflammatory (ESR and CRP) parameters also normalized after 3–4 months. Canakinumab was administered twice at 3-month intervals. Thereafter, the intervals were prolonged to every 6 months since disease activity was minimal (BSA between 2 and 4%). Usually at the end of each 6-month period, mild urticarial rashes and night sweats reappeared, but immediately disappeared within 1–2 days after the injection of canakinumab. Therapy using canakinumab 150 mg every 6 months is ongoing since over 10 years with no adverse effects, especially during the coronavirus disease 2019 (COVID-19) pandemic.

## Methods

To investigate the effect of canakinumab, we performed an immunohistochemical analysis of the cellular infiltrate from a skin biopsy specimen obtained before and 1 month after initiating treatment with canakinumab using the avidin–biotin complex-alkaline phosphatase method with the following primary antibodies: CD1a (clone 10; DakoCytomation, Glostrup, Denmark), CD4 (clone MT310, DakoCytomation), CD8 (clone C8/144B; DakoCytomation), CD11c (clone KB90, DakoCytomation), CD32 (KB61, Dakocytomation), CD68 (clone EBM11, Dakocytomation), CD163 (clone EDHU-1; Serotec MCA, Oxford, United Kingdom), CD206 (clone 19.2, BDPharmingen, Allschwil, Switzerland), inducible nitric oxide synthase (iNOS; clone EPR16635, Abcam, Cambridge, United Kingdom), tryptase (clone AA1, DakoCytomation), neutrophil elastase (clone NP57, DakoCytomation), IL-1β (clone 11E5; Abcam), IL-8 (clone 807; Abcam), and IL-17 (polyclonal, AF-317-NA, R&D Systems, Minneapolis, MN, United States). Irrelevant IgG subclass-matched antibodies were used as negative control. Quantitative analysis of positively stained cells was performed using the digital image analysis system NIS-Elements Software BR 2.30 (Nikon, Tokyo, Japan).

## Results

**Figure 2:**
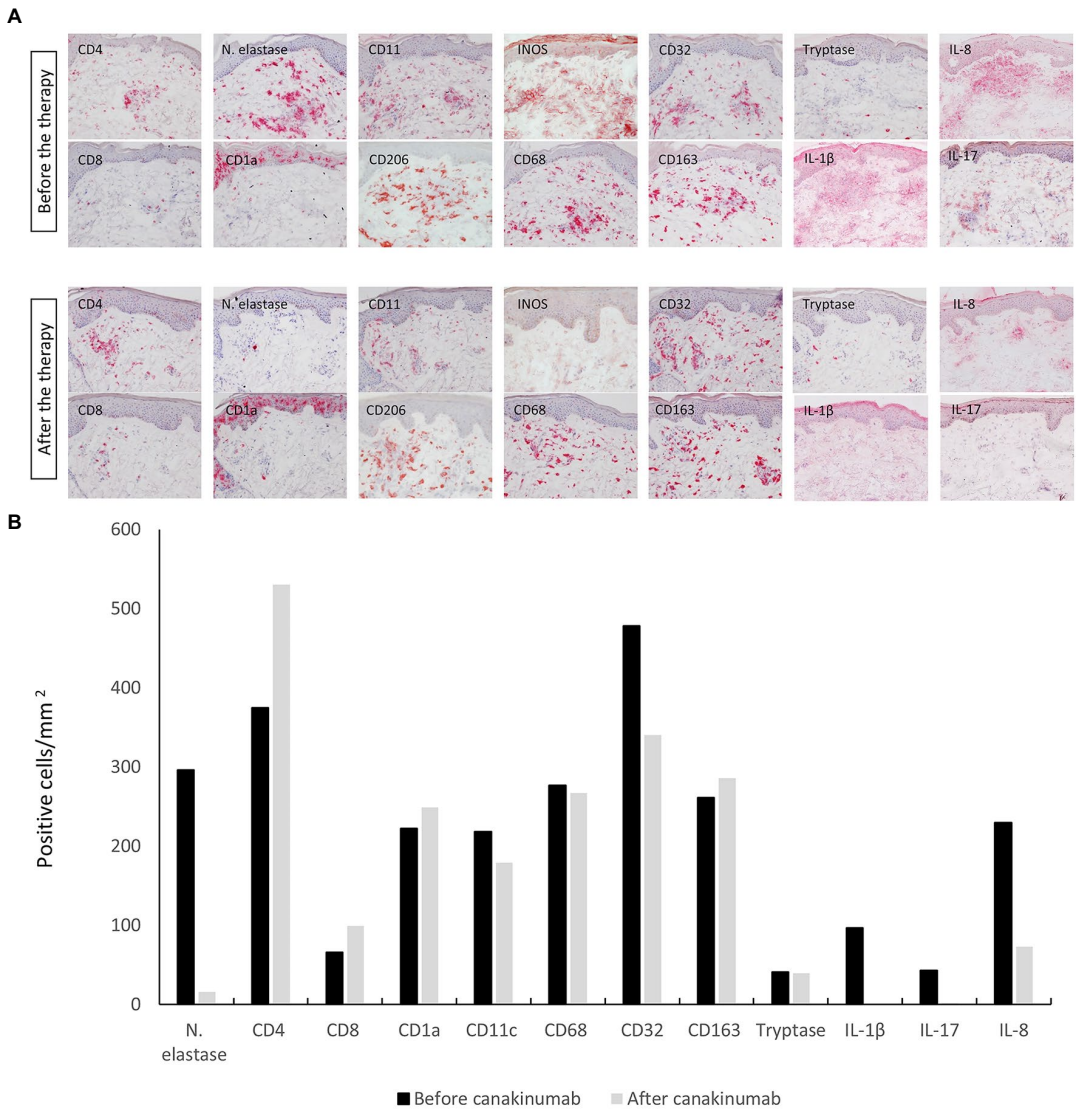
**(A)** Immunohistochemical staining of the cellular infiltrate with different leucocyte populations and proinflammatory cytokines from a skin-biopsy specimen obtained before and 1 month after initiating treatment with canakinumab. Original magnification 200 ×. **(B)** Quantification of the number of stained cells in the infiltrate (cells/mm^2^) before and 1 month after the initiation of canakinumab.

Immunohistochemical analysis showed a marked decrease in neutrophil elastase, a marker for neutrophil granulocytes, within 1 month after the first canakinumab injection. Furthermore, a substantial decrease in the expression of the proinflammatory cytokines IL-1β, IL-8, and IL-17 was observed. The latter are involved in neutrophil recruitment and activation. With regard to the dermal inflammatory infiltrate, there was a marginal decrease in cells positive for the markers of myeloid dendritic cells (CD11c^+^) and macrophage subsets, i.e., M1-like (iNOS^+^, CD68^+^, and CD32^+^) macrophages. In contrast, a slight increase was seen in Langerhans cells (CD1a^+^) and M2-like (CD206^+^ and CD163^+^) macrophages as well as for both T cell subsets (CD4^+^ and C8^+^). No difference was noted for tryptase-positive mast cells after canakinumab administration (Figures 2A,B).

## Discussion

Our observation illustrates the long-lasting sustained positive effect of canakinumab for over 10 years in a patient with Schnitzler syndrome, confirming the key role of blocking IL-1β in the disease management.

IL-1β is a proinflammatory cytokine that not only acts as a mediator of the peripheral immune response to infections and inflammation but also plays an important role in acute and chronic autoimmune diseases, diabetes, pain, and neurological disorders. A dysregulated activity of IL-1β is characteristic in autoinflammatory diseases, which may be caused either due to abnormally elevated cytokine levels or a qualitative or quantitative deficiency of the endogenous antagonist of IL-1 receptor type 1 (4).

In cryopyrin-associated periodic syndromes, there is an uncontrolled activation of caspase-1 and subsequent abnormal IL-1β secretion by a mutation in the cryopyrin-coding gene for nucleotide-binding domain, leucine-rich repeat containing gene family, pyrin domain containing protein 3 (*NLRP3*). In patients with Schnitzler’s syndrome, in whom *NLRP3* somatic mosaicism has been anecdotally identified, IL-1β secretion is up-regulated (5). The latter is thought to directly contribute to the clinical manifestations (6). In line with this notion, we observed a strong staining for IL-1β as well as for IL-8 and IL-17 together with a dense infiltrate particularly of neutrophils, dendritic cells, and macrophages in the skin lesion of our patient.

Canakinumab was developed as a human IgGκ monoclonal antibody targeting IL-1β for the treatment of immune and autoinflammatory disorders. This specific inhibition of IL-1β efficiently suppresses the neutrophil-driven inflammation and often results in a quick and effective reduction of disease activity (4, 7). We were able to demonstrate this mechanism clinically and immunohistochemically with a remarkable decrease in the proinflammatory cytokines 1β, IL-8, IL-17 and particularly of neutrophils in the skin lesions within 1 month after initiation of canakinumab. This effect has now lasted for over 10 years and the therapy is well tolerated without any side effect.

Canakinumab is generally well tolerated; however, upper respiratory tract infections are known to be the most common side effect during therapy (7, 8). In our case, the therapy with canakinumab was continued during the COVID-19 pandemic, as IL-1β together with IL-6 and tumor necrosis factor-α plays an important role as key interleukin in SARS-CoV-2-induced cytokine storm. The latter accounts for a significant part of the negative consequences of SARS-CoV-2 infection (9). Therefore, inhibition of IL-1β may be able to inhibit this excessive immune response (9, 10).

Approximately 15% of all cases of Schnitzler syndrome develop a lymphoproliferative disorder, such as Waldenström macroglobulinemia (11). Our patient showed a decrease in IgM gammopathy to 4 g/l after 1 year. Over the past years, stable values between 3.6 and 4 g/l were observed.

In brief, our case provides evidence for an excellent long-term efficacy of canakinumab in Schnitzler syndrome. There was no impact on the MGUS or its potential progression into a higher-grade lymphoproliferative disease as well as no increased susceptibility to infections. Follow-up of a large cohort of patients with Schnitzler syndrome is however necessary to better assess the response and safety of canakinumab in the long-term management of this condition.

## Data availability statement

The datasets presented in this article are not readily available because of ethical/privacy restrictions. Requests to access the datasets should be directed to the corresponding author.

## Ethics statement

Ethical approval was not provided for this study on human participants because Ethical review and approval was not required for the study on human participants in accordance with the local legislation and institutional requirements. Written informed consent was obtained from the patient for the publication of the images or data included in this article. The patients/participants provided their written informed consent to participate in this study.

## Author contributions

SB, SS, KH, KY, LF, LB, and NY designed the study and performed the acquisition, analysis, and interpretation of data. SB, SS, and NY wrote the manuscript. KH, KY, LF, and LB performed critical revision of the manuscript. All authors contributed to the article and approved the submitted version.

## Funding

This work was supported by the Department of Dermatology, Bern University, Bern, Switzerland.

## Conflict of interest

NY has served as a consultant for Novartis.

The remaining authors declare that the research was conducted in the absence of any commercial or financial relationships that could be construed as a potential conflict of interest.

## Publisher’s note

All claims expressed in this article are solely those of the authors and do not necessarily represent those of their affiliated organizations, or those of the publisher, the editors and the reviewers. Any product that may be evaluated in this article, or claim that may be made by its manufacturer, is not guaranteed or endorsed by the publisher.
